# Acute correction of severe complex adolescent late-onset tibia vara by minimally invasive osteotomy and simple circular fixation: a case series with 2-year minimum follow-up

**DOI:** 10.1186/s12891-021-04496-y

**Published:** 2021-08-12

**Authors:** Abo Bakr Zein, Ahmed S. Elhalawany, Mohammed Ali, Gerard R. Cousins

**Affiliations:** 1 Department of Orthopaedic Surgery, Faculty of Medicine, Cairo University, P.O 11562, Cairo, Egypt; 2 Present Address: Raigmore Hospital, Inverness, Scotland, UK; 3 Trauma and Orthopaedics, Health Education Northeast, Newcastle, UK

**Keywords:** Tibia vara, Proximal tibial osteotomy, Circular external fixation, Complex severe varus deformity, Blount’s disease, Acute correction

## Abstract

**Background:**

Despite multiple published reviews, the optimum method of correction and stabilisation of Blount’s disease remains controversial. The purpose of this study is to evaluate the clinical and radiological outcomes of acute correction of late-onset tibial vara by percutaneous proximal tibial osteotomy with circular external fixation using two simple rings. Weighing up the pros and cons and to establish if this method would be the method of choice in similar severe cases especially in a context of limited resources.

**Methods:**

This study was conducted between November 2016 and July 2020. We retrospectively reviewed the clinical notes and radiographs of 30 patients (32 tibiae) who had correction of severe late-onset tibia vara by proximal tibial osteotomy and Ilizarov external fixator. The mean age at the time of the operation was 16.6 (± 2.7) years (range 13–22).

**Results:**

The mean proximal tibial angle was 65.7° (± 7.8) preoperatively and 89.8° (± 1.7) postoperatively (*p* < 0.001). The mean mechanical axis deviation improved from 56.2 (± 8.3) preoperatively to 2.8 (± 1.6) mm postoperatively (*p* < 0.001). The mean femoral-tibial shaft angle was changed from –34.3° (± 6.7) preoperatively to 5.7° (± 2.8) after correction, with degree of correction ranging from 25° to 45°. Complications included overcorrection (three cases 9%) and pin tract infection (eight cases 25%). The mean Hospital for Special Surgery knee scoring system (HSS) improved from 51.03 (± 11.24) preoperatively to 94.2 (± 6.8) postoperatively (*p* < 0.001). The mean length of follow up period 33.22 (± 6.77) months, (rang: 25–46 months). At final follow up, all patients had full knee range of motion and normal function. All cases progressed to union and there were no cases of recurrence of deformity.

**Conclusion:**

This simple procedure provides secure fixation allowing early weight bearing and early return to function. It can be used in the context of health care systems with limited resources. It has a relatively low complication rate. Our results suggest that acute correction and simple circular frame fixation is an excellent treatment choice for cases of late-onset tibia vara, especially in severe deformities.

## Background

Idiopathic tibia vara or Blount’s disease has been categorised into three groups based on age-onset. These are; infantile group which is less than 3 years, juvenile which is 4 to 10 years and adolescent group which is 11 years or older [1]. Blount’s disease results in a complex three-dimensional deformity namely varus, internal tibial torsion and procurvatum [2–5]. These deformities result in gait abnormalities, limb-length discrepancy, early onset osteoarthritis and as concomitant deformities in the distal femur and distal tibia [6–8]. Addressing these deformities requires very detailed and careful planning in order to achieve satisfactory results and avoid potential complications [9, 10]. The correction main aim is to reduce the abnormal stresses on the knee joint so as to prevent the occurrence of osteoarthritis and any future functional disability [10, 11].

Although Blount’s disease has been thoroughly studied with multiple published reviews and reports, the method of correction and stabilization remains controversial. Several protocols have been published describing different techniques of acute correction using either internal or external fixation methods [12–18]. The use of Ilizarov external fixators and Taylor spatial frames in order to achieve gradual corrections is well established [11, 12, 19]. When dealing with late onset sever deformities, the majority of the published literature advocates using proximal tibial osteotomy for correction regardless of the fixation method [11, 12, 15, 20–23]. There is very little evidence to recommend one form of correction over the other [24].

The aim of this study is to evaluate the clinical and radiological outcomes of the acute correction of late onset tibial vara using percutaneous proximal tibial osteotomy with circular external fixation by simple two rings and weighing up the pros and cons to establish if this method would be the method of choice in similar cases.

## Methods

We retrospectively reviewed the clinical notes and radiographs of 48 consecutive patients who had correction of late-onset tibia vara and completed their 2-year follow up between November 2016 and July 2020. The study was conducted in the Limb Deformity Reconstruction unit of Cairo University Hospital. Written informed consent was obtained from all participants. Included patients presented with severe late-onset tibia vara deformity defined as 20° or more angulation on the coronal plane between the femoral and the tibial shafts (i.e., a femoral–tibial shaft angle (F-T angle) of ≥ 20° varus). Patients with a depressed medial tibia plateau, patients younger than 13 years and those with abnormal lateral distal femoral angle were excluded. Examination, deformity analysis and radiological evaluations of the lower limbs were performed for all patients by single-independent investigator (AS). All patients had a single stage correction by proximal tibial osteotomy distal to tibial tuberosity and two rings Ilizarov external fixation (IEF). Data was collected at baseline, immediately after correction and at final follow up. We utilised standard weight-bearing anteroposterior and lateral radiographs of both lower extremities in addition to CT scanographs to measure the mechanical axis deviation, F-T angle, medial proximal tibial angle and posterior proximal tibial angle. The foot-thigh angle was measured clinically. We used the Hospital for Special Surgery (HSS) knee scoring system for the clinical evaluation of our patients pre- and postoperatively [25].

### Surgical technique

Pre-operatively, all patients had their deformities measured and assessed clinically and radiologically. Corrections were templated by the senior author (AZ). Incision site, osteotomy orientation and the construction of the two-ring Ilizarov external fixator frame were all planned pre-operatively. The frame consisted of two symmetrical rings connected by four connecting threaded rods. Schanz clamps and Rancho cubes were then added to the frame according to the planned Schanz screws sites.

**Fig. 1:**
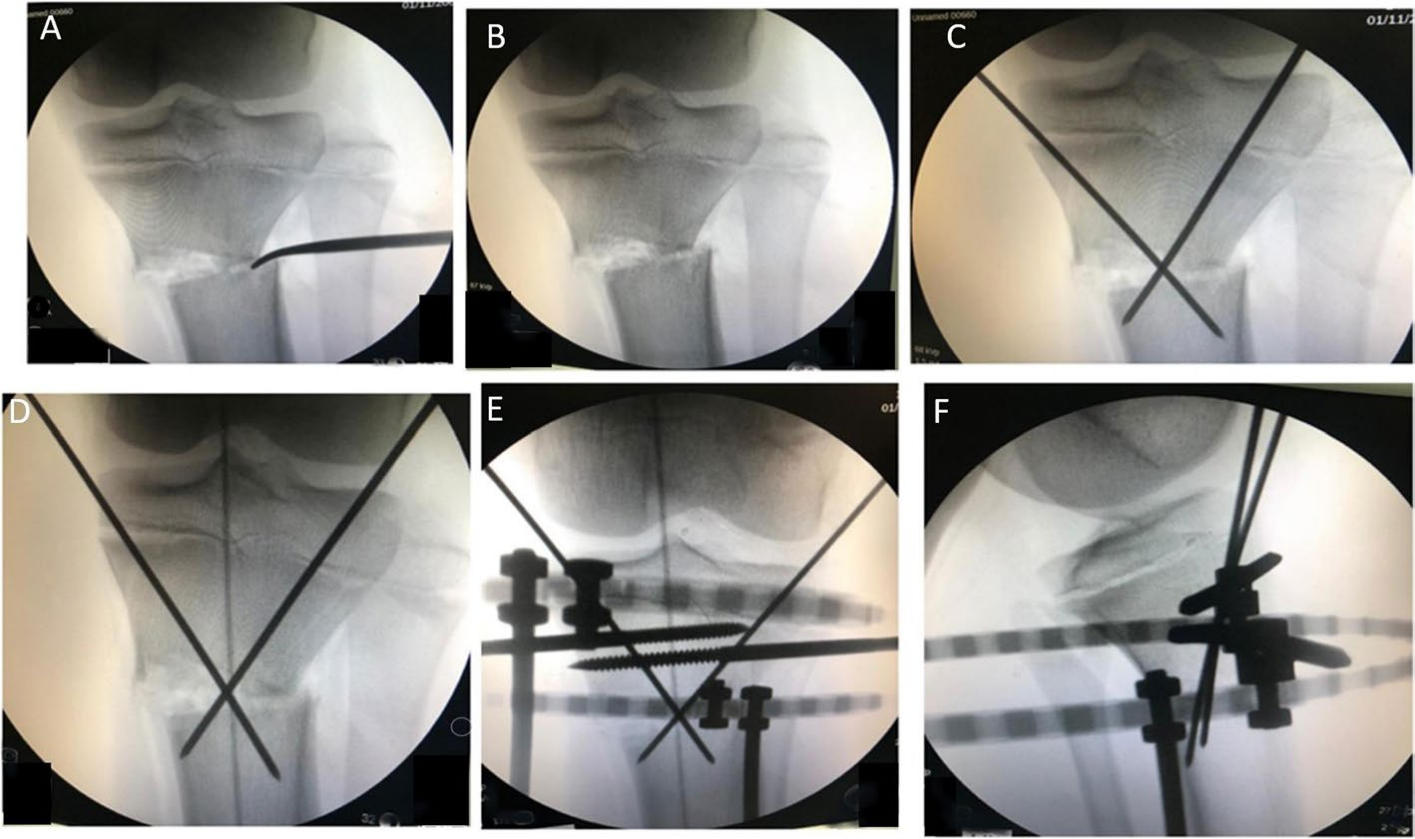
Intraoperative C-arm views showing the various steps of the procedure: **A** A Hohmann retractor was used through the osteotomy site as a lever to maintain the correction and lateral translation of the distal segment. **B** Acute multiplanar correction was then carried out which included derotation, angulation, translation. **C** Two converging 2 mm wires were used for the preliminary fixation of the osteotomy. **D** Assessment of the correction by the mechanical access highlighted by the shadow of the electrocautery cable passes through the centre of the knee. **E**, **F** Anteroposterior and lateral views during application of the frame

**Fig. 2:**
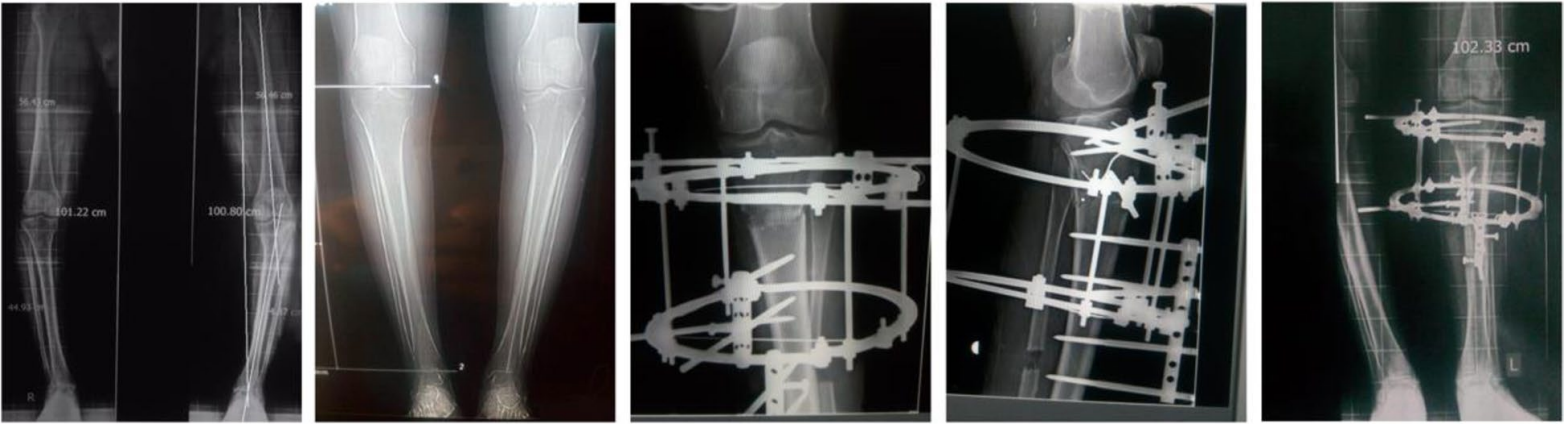
Pre- and post-operative radiographs showing acute correction of late onset tibia vara in 22-year-old male patient

Surgery was performed under either general or spinal anaesthesia based on patient’s age and fitness. Patients were positioned supine, tourniquet was not used, and a broad-spectrum intravenous antibiotic was given on induction of anaesthesia. The whole operated limb was prepared and draped from the anterior superior iliac spine (ASIS) to the toes in order to facilitate the intraoperative alignment check and allow room for the image intensifier. Fibular osteotomy was performed through a 3 cm lateral skin incision at the level of the middle third of the fibula. Multiple drill holes were made in the fibula over an area of 2 cm and osteotomy completed. Percutaneous straight corticotomy was performed though a vertical anterior 3 cm-incision distal to the tibial tuberosity. This was followed by multiple drill holes using a 4.5 mm drill bit. The osteotomy cut was completed using an osteotome. Acute multiplanar correction was then carried out which included derotation, angulation, translation and extension if required. A Hohmann retractor was used through the osteotomy site as a lever to maintain the correction and lateral translation of the distal segment. The correction was assessed clinically and radiologically using image intensifier (Fig. 1). A cable was stretched from the centre of the femoral head to the centre of the ankle joint aiming for overcorrection (passed through centred of the knee slightly through its lateral compartment). The joint orientation line was checked on the AP view. The lateral view was used to check the tibial slope and the procurvatum correction. Two converging 2 mm wires were used for the preliminary fixation of the osteotomy (Fig. 1). After achieving a satisfactory correction, Ilizarov frame (two full rings) was then applied to stabilise the correction. The patella was directed to face forward under image intensifier, then a proximal reference Ilizarov wire or a Schanz screw was inserted parallel to the knee joint orientation line and just distal to the proximal tibial epiphyseal plate from lateral to medial. The proximal ring was then attached to it. Three to four 6 mm Schanz screws were then added in each ring in different planes and directions (respecting the safe zones) through Rancho cubes or Schanz clamps (Fig. 2). At the end, the final position was rechecked clinically and with the image intensifier and the skin closed with simple interrupted sutures.

Postoperatively, the neurovascular status was checked, patients were encouraged to actively exercise the knee and to weight bear as tolerated with crutches. Patients were reviewed by our physiotherapy team, instructed on wound and pin sites care and discharged on the second postoperative day. After 2 weeks, patients presented for wound check and removal of the stitches. Four to 6 weeks later assessment of correction, union, function and complications were carried out clinically and radiologically, and further follow-up arranged accordingly. The IEF was dynamized when there was radiological evidence of union. The IEF was then removed completely when full union was achieved clinically and radiologically. In our practice this was generally 2–3 weeks following the dynamization.

### Statistics

Statistical analysis was performed using SPSS (Statistical Package for the Social Sciences) version 26 (IBM, Armonk, New York, USA). Data was summarized using the mean and the standard deviation (SD) for quantitative variables and frequencies (count) and relative frequencies (percentages) for categorical variables.

Continuous variables included age, preoperative/postoperative deformity angles, Mechanical axis deviation (MAD), length of follow up period, bone union time. Categorical variables were sex, side and complications. Comparisons between preoperative and postoperative variables were done using paired t test [26]. *P*-values less than 0.05 were considered as statistically significant*.*

## Results

Eighteen patients were excluded as they did not meet the inclusion criteria or were lost to follow up. Therefore, the study included 32 tibiae (30 patients), 26 males (86.7%) and 4 females (13.3%). The mean age at the time of the operation was 16.6 (± 2.7) years (range 13–22). The right side was affected in 14 patients (44%) and the left sided was affected in 18 patients (56%). The mean duration of Ilizarov was 12.6 (± 2.3) weeks (range from 10 – 18 weeks).

**Fig. 3:**
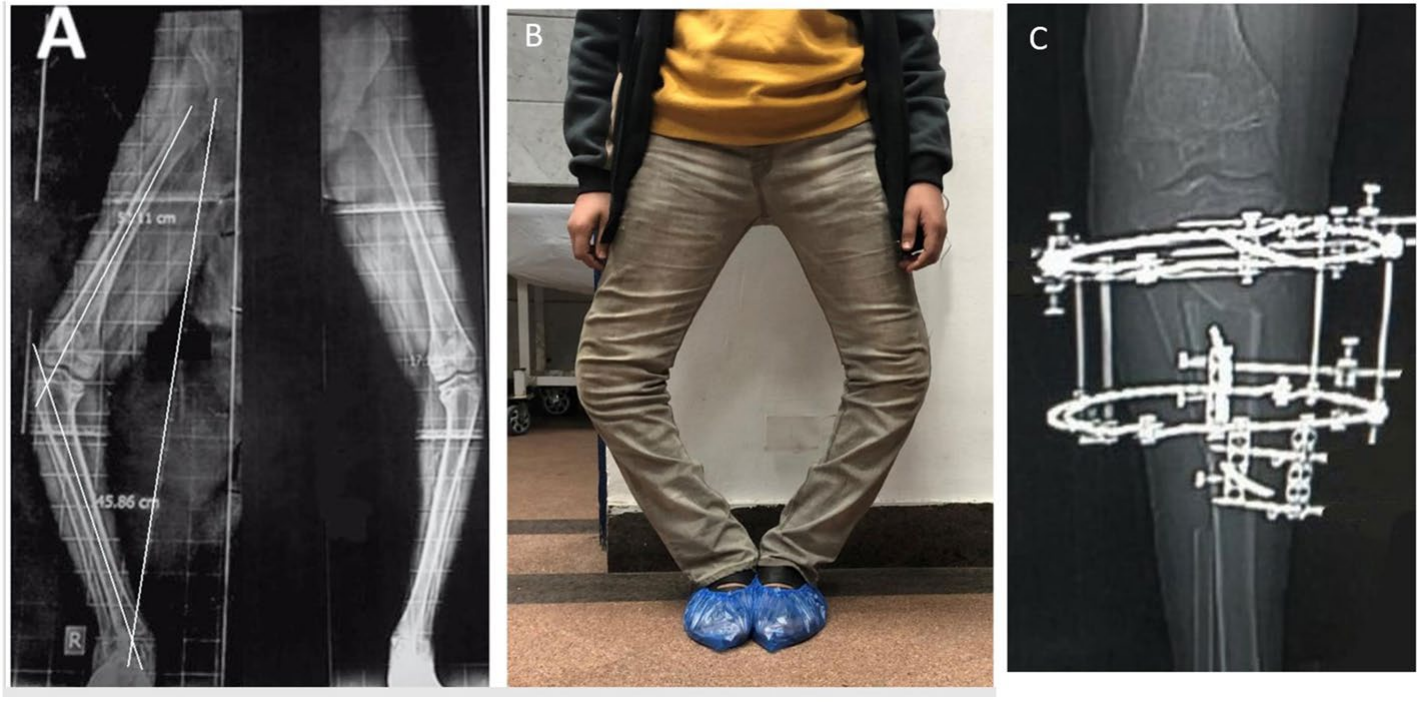
Preoperative full leg length scanogram (TFA -48°) (**A**) and photograph (**B**) of a 15-year-old male with bilateral severe late-onset tibia vara and ankle valgus. **C** Post-operative radiograph after knee deformity correction showing the osteotomy

**Fig. 4:**
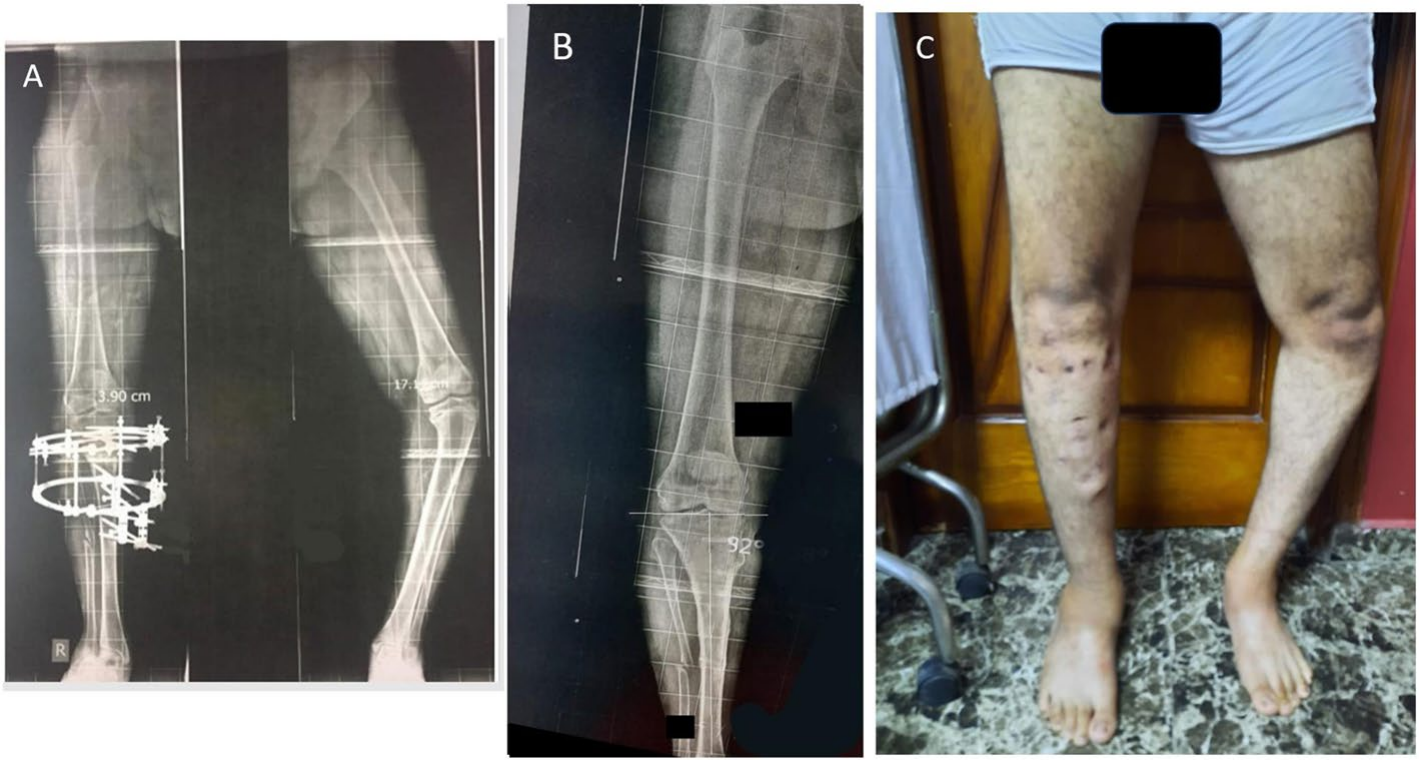
Post-operative scanogram 3 months follow-up before Ilizarov frame removal with normalization of the mechanical axis (**A**). Radiograph (fully united) and clinical appearance after Ilizarov frame removal 3.5 months of the previously prescribed patient (**B**, **C**)

The mean proximal tibial angle was 65.7° (± 7.8) preoperatively and 89.8° (± 1.7) postoperatively (*p* < 0.001). The mean MAD improved from preoperatively 56.2 (± 8.3) to 2.8 (± 1.6) mm postoperatively (*p* < 0.001). The mean Femoral-tibial shaft angle was changed from –34.3° (± 6.7) preoperatively to 5.7° (± 2.8) after correction (*p* < 0.001), and the range of angular correction was from 25° to 45° (Table 1; Figs. 3 and 4). The mean preoperative HSS score of our patients was 51.03 (± 11.24). Postoperatively the average HSS knee score was 94.2 (± 6.8). Excellent or good results were present in 91% of cases and fair in 9%. HSS score improved by an average of 42 points (range: 14–58 points) and this improvement was statistically significant (*p* < 0.001) (Table 1). All patients in this study maintained near full range of motion of the knee during frame application. The range of motion of the knee was not decreased in any patient at the latest follow up.

**Table 1:** Measurements pre and postsurgical correction

	**Preoperative (Mean ± SD)**	**Postoperative (Mean ± SD)**	*P* Value*
Mechanical axis deviation **(MAD)**	56.2 (± 8.3) mm	2.8 (± 1.6) mm	< 0.001
Femoral–tibial shaft angle **(FTA)**	–34.3° (± 6.7)	5.7° (± 2.8)	< 0.001
Medial proximal tibial angle **(MPTA)**	65.7° (± 7.8)	89.8° (± 1.7)	< 0.001
Posterior proximal tibial angle **(PPTA)**	72.7° (± 3.1)	80.4° (± 1.1)	< 0.001
Thigh-foot angle **(TFA)**	–18.5° (± 4.8)	10° (± 3.5)	< 0.001
Knee Score	51.03 (± 11.24)	94.2 (± 6.8)	< 0.001

^*^ Paired t test for comparison between pre- and post-operative values

Regarding complications, eight patients (25%) developed one or more episode of superficial pin site infection. This was mainly reported around the proximal Schanz screws or wires. All cases improved following local pin site care and oral antibiotics except for one patient who developed deep pin site infection and required formal washout and changing the sites of the loose Schanz screws. There were no cases of septic arthritis of the knee, osteomyelitis, fractures, neurovascular injuries, compartment syndrome, loss of the alignment or non-union. Overcorrection was noted in three cases. One was readjusted immediately after surgery, the second was done 2 weeks post-operatively and the last case did not require readjustment as did not affect function or patient satisfaction.

All cases completed 2 years of follow up. The mean length of follow up period 33.22 (± 6.77) months, (rang: 25 – 46 months). At the final review, all the patients had full knee range of motion and function, the osteotomies were fully united and no further deformity had developed.

## Discussion

In this paper we report the clinical and radiological outcomes following single stage correction of severe tibia vara deformities. The study selected patients with severe deformities the majority of whom presented at a late stage, often coming from rural and peripheral areas where the health services are deficient. This disease varies in its severity, morphology, concomitant deformities, age and time of presentation, and there is no gold standard correction technique or fixation method [10–15].

Based on the published literature, the external fixator is a popular fixation tool that can be easily applied to stabilise the bone after both angulation and translation and allows for early weight bearing. This promotes both soft tissue and bone healing. In addition, it is applied percutaneously without periosteal disruption. Furthermore, an external fixator is adjustable and any residual degree of under or overcorrection can be managed through the device at the bedside or at the early outpatient follow-up reviews [14–16, 27].

Our results are in agreement with Ashfaq’s [28] who suggested using the monolateral frame in mild proximal tibial vara deformities of less than 10 degrees and the spatial frame for deformities of more than 10 degrees or when there are concomitant sagittal or axial deformities. While Dammerer et al. [29] reported more accurate results using TSF compared to IEF, we used the IEF as a fixed construct applied after deformity correction and preliminary fixation. Clearly, circular fixators give more secured fixation than mono planar external fixator as Schanz screws can be applied through different axes allowing better fixation of the short proximal tibial fragment following the osteotomy. We would argue it’s multi-planar nature makes a circular frame more secure and certainly more adjustable than internal fixation. Furthermore, having a complete correction in one setting and the use of simple IEF are cost-effective for both patients and the health service.

There have been reported complications in relation to acute correction and circular frames which include transient and permanent neurological damage [4, 16, 30]. We have not experienced any of these complications in this series and we believe careful dissection and respect of safe zones should avoid such complications. When inserting Shanz screws our aim is to get it right first time. Multiple passes and moving the screws around can cause loosening which may be a cause for failure especially in the proximal metaphyseal part. Applying this concept, we found that the circular external fixator allows best hold of the short proximal fragment and gives more secured fixation. We did not experience any case of loosening or loss of position and alignment.

Pin tract infection was the most frequent complication we encountered however only one case required a formal debridement. In our series we tried to avoid extreme degree of overcorrection. There were three cases of overcorrection and no cases of under-correction or recurrence were recorded. Overcorrection has been a common practice by most orthopaedic paediatric surgeons in order to prevent recurrence of deformity [31]. Eamsobhana et al. [31] shared the same views and their study showed the overcorrection group had no statistically significant recurrence rate compared to the non-overcorrection group, and overcorrection more than valgus 15° has no benefit to prevent recurrence.

The severity of the deformity can be a major concern when planning correction however in our experience, all the deformity components can be corrected efficiently with the described methods without any concerns.

## Study limitations

Retrospective study and small number of patients. The duration of follow obviously does not allow assessment of delayed complications, particularly degenerative change such as knee osteoarthritis, recurrence of the varus malignment or ligamentous laxity. It would be interesting to investigate if prior tibia vara surgery predisposes to the outcome of knee arthroplasty.

## Conclusion

The correction and fixation method of Blount’s disease are decided on an individual basis as it depends on the severity of the deformity and the surgeon preference. The technique we have described results in a fast recovery time, good surgical outcomes and fewer complications. It provides rigid fixation, allowing early weight bearing and early restoration of limb function. The resulting early weight bearing promotes both soft tissue and bone healing. This method can be used in settings with limited resources and implants. With the ongoing debate as to optimal treatment, we suggest single-stage correction and simple circular frame fixation, especially in the severe deformities should be the surgical technique of choice.

## Data Availability

The datasets used and analysed during this study are available from the corresponding author on reasonable request.

## Abbreviations

ASIS: Anterior superior iliac spine.
CT: Computerized tomography
F-T angle: Femoral–tibial shaft angle
HSS: Hospital for Special Surgery knee scoring system
IEF: Ilizarov external fixation
MAD: Mechanical axis deviation
MPTA: Medial proximal tibial angle
PPTA: Posterior proximal tibial angle
SPSS: Statistical Package for the Social Sciences
TSF: Taylor spatial frame
